# Vaccination with Ectoparasite Proteins Involved in Midgut Function and Blood Digestion Reduces Salmon Louse Infestations

**DOI:** 10.3390/vaccines8010032

**Published:** 2020-01-19

**Authors:** Marinela Contreras, Marius Karlsen, Margarita Villar, Rolf Hetlelid Olsen, Lisa Marie Leknes, Anette Furevik, Karine Lindmo Yttredal, Haitham Tartor, Soren Grove, Pilar Alberdi, Bjorn Brudeseth, José de la Fuente

**Affiliations:** 1 SaBio, Instituto de Investigación en Recursos Cinegéticos IREC-CSIC-UCLM-JCCM, Ronda de Toledo s/n, 13005 Ciudad Real, Spain; marinela.contreras@uclm.es (M.C.); margaritam.villar@uclm.es (M.V.); maria.alberdi@uclm.es (P.A.); 2 Pharmaq AS, P.O. Box 267, Skoyen, N-0213 Oslo, Norway; marius.karlsen@zoetis.com (M.K.); Rolf.Hetlelid-Olsen@zoetis.com (R.H.O.); lisa-marie.leknes@zoetis.com (L.M.L.); Anette.Furevik@zoetis.com (A.F.); karine.yttredal@zoetis.com (K.L.Y.); 3 Biochemistry Section, Faculty of Science and Chemical Technologies, and Regional Centre for Biomedical Research (CRIB), University of Castilla-La Mancha, 13071 Ciudad Real, Spain; 4 Norwegian Veterinary Institute, 0106 Oslo, Norway; haitham.tartor@vetinst.no (H.T.); soren.grove@vetinst.no (S.G.); 5 Institute of Marine Research, 5005 Bergen, Norway; 6 Department of Veterinary Pathobiology, Center for Veterinary Health Sciences, Oklahoma State University, Stillwater, OK 74078, USA

**Keywords:** salmon louse, immunology, vaccinology, copepod, aquaculture

## Abstract

Infestation with the salmon louse *Lepeophtheirus salmonis* (Copepoda, Caligidae) affects Atlantic salmon (*Salmo salar* L.) production in European aquaculture. Furthermore, high levels of salmon lice in farms significantly increase challenge pressure against wild salmon populations. Currently, available control methods for salmon louse have limitations, and vaccination appears as an attractive, environmentally sound strategy. In this study, we addressed one of the main limitations for vaccine development, the identification of candidate protective antigens. Based on recent advances in tick vaccine research, herein, we targeted the salmon louse midgut function and blood digestion for the identification of candidate target proteins for the control of ectoparasite infestations. The results of this translational approach resulted in the identification and subsequent evaluation of the new candidate protective antigens, putative Toll-like receptor 6 (P30), and potassium chloride, and amino acid transporter (P33). Vaccination with these antigens provided protection in Atlantic salmon by reducing adult female (P33) or chalimus II (P30) sea lice infestations. These results support the development of vaccines for the control of sea lice infestations.

## 1. Introduction

Blood feeding arthropod ectoparasites affect a variety of species and can transmit pathogens, causing diseases in humans and animals worldwide [1]. Infestation with the salmon louse *Lepeophtheirus salmonis* (Copepoda, Caligidae) is currently one of the main constraints for further growth of Atlantic salmon (*Salmo salar* L.) production in European aquaculture [2]. Although mortality in farmed fish occurs only in extreme cases, the salmon louse and other parasitic copepods affect the host negatively by reducing immune competence, thus making it more susceptible to other infections [3,4]. The salmon louse is also known to be a vector of fish pathogens [5,6].

The salmon louse lifecycle consists of eight instars that are separated by molting events [7,8]. After hatching, lice go through two pelagic naupliar stages. These stages are not infective and last long enough to transport the larvae over significant distances with sea currents. The infective copepodid stage then attaches specifically to salmonid hosts and transforms into the two chalimus stages. These stages are attached to the host through an anchor-like structure and do not jump between hosts. The last chalimus stage molts into the two pre-adult stages that are fully motile and may jump between nearby hosts. Transition into adult stages then occurs, where fertilized females develop several sets of eggstrings. The eggstrings are usually attached to the female until hatching when a new generation of nauplia is released to the surroundings. Feeding occurs in all on-host stages. While the younger instars mainly feed on mucus and skin, blood-feeding is observed in pre-adult lice. The adult females are considerably larger than the males and feed almost exclusively on blood.

Farmed populations of salmon are the main reservoir of *L. salmonis* [9]. High levels of salmon lice in farms, therefore, significantly increase challenge pressure against wild salmon populations. As a result, regulators in salmon producing countries have enforced strict limitations to the allowed sea lice levels in a farm. These regulations have led to frequent treatments with pesticides, most of which now have lost efficacy due to reduced sensitivity [10]. Other methods based on more mechanical solutions, such as temperature shock, freshwater bath, and flushing the surface of the fish with pressurized water, have emerged [11,12]. Although efficacious, these methods involve stressful handling and have welfare issues for the fish. Many salmon producers are also cultivating various species of cleaner fish (family *Labridae* and lumpsuckers *Cyclopterus lumpus*) together with the salmon [13,14]. The sole purpose of these fish is to feed on salmon lice. This co-cultivation does indeed lead to lower levels of adult salmon lice, but the mortality among the cleaner fish is high, thus raising ethical debates. Given the challenges with currently available methods, vaccination appears as an attractive, environmentally sound strategy. Even a relatively moderate level of vaccine efficacy could be a useful tool for salmon lice control since most of the hosts that produce new generations of lice are under human control, and vaccine coverage in the total host population could, therefore, be high. However, no commercially available anti-sea lice vaccines exist. Previously published results suggest that the discovery of protective antigens, vaccine formulation, safety, administration, and efficacy need to be addressed to obtain regulatory approval and advance in the implementation of vaccination strategies for the control of salmon lice and other fish ectoparasites [15,16,17,18,19,20].

Ectoparasite–host–pathogen interactions have evolved through dynamic processes involving genetic traits of hosts, pathogens, and ectoparasites that mediate their development and survival [21]. New approaches for ectoparasite control are dependent on defining molecular interactions between hosts and ectoparasites to allow for the discovery of key molecules that could be tested in vaccines for the intervention of ectoparasite-host cycles [21,22]. Research on ticks has led this area, and tick vaccines based on recombinant BM86/BM95 antigens are the only commercially available vaccines against ectoparasites [20,21,23,24]. These vaccines offer the important advantages of being a cost-effective and environmentally friendly alternative to ectoparasite control with a dual effect of reducing tick infestations and preventing ticks from transmitting disease-causing pathogens. Tick vaccine research provides models for the development of vaccines against other arthropods, such as mosquito, sand fly, dust mite, poultry red mite, Australian sheep blowfly, and sea louse with the possibility of controlling multiple ectoparasite infestations [22,23,24,25]. The challenge of developing vaccines against ectoparasites arises from the need to understand the complex molecular interactions between vertebrate hosts and ectoparasites, which require the discovery of key pathway molecules that mediate ectoparasite–host interactions [20,26].

In this study, we targeted the salmon louse midgut function and blood digestion for the identification of candidate target proteins for the control of salmon louse infestations. In this translational approach, basic biological information on ectoparasite–host interactions translates into the identification and subsequent evaluation of new candidate protective antigens.

## 2. Materials and Methods

### 2.1. Salmon Lice and Tissues

Salmon lice (*L. salmonis*) of the strain Ls. Gulen [27] were purchased from Industrilaboratoriet in Bergen (ILAB, Norway) and used for all experiments, either as copepodids, pre-adults, or adult females. Intestinal tissues from fed and starved adult females were obtained by dissection of live lice fed with blood in intestine that had been recently picked off the Atlantic salmon host, or live lice starved with no visible blood in intestine that had been kept for approximately 3 days off the host in seawater. Only females were used because of their key role in louse fecundity and their largest bloodmeal when compared to males. The isolation of the intestine was done in seawater under a loupe using a scalpel and tweezers to remove as much superfluous tissue as possible. Isolated intestines were then stored frozen (−80 °C) in RNAlater (Sigma–Aldrich, St. Louis, MO, USA) for analyses of mRNA levels or in PBS for protein analysis.

### 2.2. Salmon Louse RNA and Protein Extraction

Total RNA was extracted from unfed and fed sea lice gut samples using Qiazol and the TissueLyzer (Qiagen, Madrid, Spain) following the manufacturer’s protocol (RNeasy^®^ Lipid Tissue Mini Kit; Qiagen) until the water phase was recovered. Then, the protocol for the RNeasy^®^ Mini Kit (Qiagen) was followed, including the DNase in-column treatment. For both samples, more than 7 μg total RNA was obtained, and as expected, the unfed:fed RNA ratio was approximately 1:3. The quality of the RNA was checked using the BioAnalyzer 2100 (Agilent Technologies, Santa Barbara, CA, USA).

A protocol was developed for the extraction of louse gut plasma membrane proteins. Frozen guts from fed (30 mg) and unfed (20 mg) *L. salmonis* were homogenized with a glass homogenizer (20 strokes) in STM solution (0.25 M sucrose, 1 mM MgCl_2_, 10 mM Tris-HCl, pH 7.4) supplemented with complete mini protease inhibitor cocktail (Roche, Basel, Switzerland) (10 mL/g tissue). The sample was sonicated for 1 min in an ultrasonic cooled bath followed by 10 sec of vortex. After 3 cycles of sonication–vortex, the homogenate was centrifuged at 260× *g* for 5 min at 4 °C to remove cellular debris. The supernatant was then centrifuged at 13,000× *g* for 30 min at 4 °C, and the pellet fraction enriched in crude plasma membranes was collected, resuspended in 150 μL STM solution supplemented with 0.7% n-Dodecyl-B-d-Maltoside (DDM) and 0.5% 3-[N,N-Dimethyl(3-myristoylaminopropyl)ammonio]propanesulfonate, Amidosulfobetaine-14 (ASB14) (detergents), incubated on a shaker 1 h at 4 °C (vortex of 5 sec after 15 min periods) and centrifuged at 13,000× *g* for 30 min at 4 °C. The soluble plasma membrane was precipitated using chloroform/methanol, dried, and stored at −80 °C until used.

### 2.3. Fish, Husbandry, and Ethics Approval

In vivo studies were conducted in compliance with approvals 6174, 8497, and 8733 issued by the Norwegian Food Safety Authority. Atlantic salmon parr were acquired from ILAB (Industri Laboratoriet, Bergen, Norway) and kept in freshwater at 12 °C. The fish were monitored daily and fed according to appetite. Vaccination for all studies was done in tanks containing 500 L freshwater. Smoltification was induced during the immunization period by giving the fish a 24 h light signal for approximately 4–6 weeks. Following transfer to seawater, the fish were challenged with copepodids or pre-adults, as described below. During challenge the fish were kept either in 500 L common tanks (test and control groups tagged and mixed in the same tank), or in a 20 L single tank system similar to that described by Hamre and Nilsen [28]. The latter model had limited statistical power but was used to measure vaccination effect on egg production and hatching to avoid the bias caused by lice jumping between hosts when test and control groups are held in the same tank.

### 2.4. Production of Recombinant Proteins and Vaccine Formulation

For the production of the recombinant proteins, *Escherichia coli* BL21 Star™(DE3) One Shot^®^ cells (Invitrogen-Life Technolgies, Inc., Grand Island, NY, USA) were transformed with the target gene cloned into the pET101/D-TOPO expression vector (Ref. K101-01, Invitrogen-Life Technologies). The entire transformation reaction was inoculated into 10 mL of Luria–Bertani (LB) containing 50 µg/mL ampicillin (Sigma–Aldrich, St Louis, MO, USA) and 0.4% glucose (Laboratorios CONDA S.A., Madrid, Spain) and kept growing overnight at 37 °C with shaking. Two milliliters of the overnight culture was propagated into 250 mL flasks containing 5LB, 50 µg/mL ampicillin and 0.4% glucose for 2 h at 37 °C and 200 rpm, and then for 4 h after addition of 0.5 mM final concentration of isopropyl-β-d-thiogalactopyranoside (IPTG, Sigma–Aldrich) for induction of gene expression. The cells were harvested by centrifugation at 3900× *g* for 15 min at 4 °C and stored at −80 °C for protein purification.

One gram of the recombinant *E. coli* cells harvested and after induction of gene expression was resuspended in 5 mL of lysis buffer (100 mM Tris–HCl, pH 7.5, 250 mM NaCl, 7 M Urea, 10 mM imidazole) containing protease inhibitor (Ref. 04693132001, Roche, San Cugat del Vallés, Barcelona, Spain) and disrupted using a cell sonicator (Model MS73; Bandelin Sonopuls, Berlin, Germany). After disruption, the insoluble protein fraction containing the recombinant antigens as inclusion bodies were collected by centrifugation at 15,000× *g* for 15 min at 4 °C and stored at −20 °C before purification. Purification of the recombinant proteins was conducted using 1 mL HisTrap FF columns mounted on an AKTA-FPLC system (GE Healthcare, Piscataway, NJ, USA) in the presence of 7 M urea lysis buffer. The eluted fraction containing the purified proteins was dialyzed against 20 volumes of PBS (137 mM NaCl, 2.7 mM KCl, 10 mM Na_2_HPO_4_, 1.8 mM KH_2_PO_4_), pH 7.4 for 12 h at 4 °C. Protein concentration was determined using bicinchoninic acid (Ref. 23225, Pierce™ BCA Protein Assay Kit, Thermo Scientific, Rockford, IL, USA). Recombinant proteins were concentrated using an Amicon Ultra-15 ultrafiltration device (cut off 10 kDa) (Millipore-Merck, Darmstadt, Germany). For vaccine formulation, recombinant proteins or saline control were adjuvanted in Montanide ISA 50 V2 (Seppic, Paris, France).

### 2.5. Polyacrylamide Gel Electrophoresis and Western Blot for the Characterization of Recombinant Proteins

The recombinant proteins were analyzed by electrophoresis and Western blot. Ten micrograms of recombinant proteins were loaded onto a 12% SDS-polyacrylamide gel (CBS scientific precast gels, Thermo Fisher Scientific, Waltham, MA, USA) and stained with Coomassie Brilliant Blue (VWR, Radnor, PA, USA). For Western blot analysis, the gel was transferred to a nitrocellulose membrane. The membrane was blocked with 5% bovine serum albumin (BSA) (Sigma–Aldrich) for 2 h at room temperature (RT), washed four times with TBS (50 mM Tris-Cl, 150 mM NaCl, 0.5% Tween 20, pH 7.5). Pooled sera collected from vaccinated salmons before salmon louse infestation at approximately week 10 post-initial vaccination were used as primary antibodies. Primary antibodies were used at a 1:200 dilution in TBS, and the membrane was incubated overnight at 4 °C and washed four times with TBS. The membrane was then incubated with mouse anti-rainbow trout antibodies (Bio-Rad Laboratories, Inc., Hercules, CA, USA) diluted 1:1000 in TBS with 3% BSA. The membrane was washed three times with TBS and incubated with a goat anti-mouse IgG-Haptoglobin-related protein (HPR) conjugate (Sigma–Aldrich). Finally, the membrane was washed three times with TBS and developed with TMB (3,3′, 5,5′-tetramethylbenzidine) stabilized substrate for HRP (Promega, Madrid, Spain) according to the manufacturer’s recommendations.

### 2.6. Fish Vaccination and Challenge Infestation with Salmon Lice

The fish were starved for one day before vaccination. On the day of vaccination, fish were anesthetized with 100 mg/L Tricain (PHARMAQ AS, Norway), tagged by shortening of adipose fins, and injected intraperitoneally with 0.1 mL of the test vaccine using a pre-calibrated syringe. Following a short recovery period, the fish were returned to the experimental tank. An identical boost vaccination was done 5 weeks after the initial injection. Challenge with lice was done around 10 weeks post the initial vaccination according to a model that is commercially available at ILAB. Briefly, the water level was lowered to approximately 15 cm depth, before around 60 lice copepodids per fish were dispersed over the surface. The tank was then slowly filled again with fresh seawater, allowing around 45 min before the tank was back at a normal level and flow-through was restored. Groups to be compared for louse levels in these trials were always kept together in the same tank to eliminate tank effects. Values in vaccinated and control groups were compared by Student’s *t*-test with Welch’s correction for unequal variances (*p* = 0.05, *n* = 35–40). The effect on males was not calculated because their contribution to fecundity is unclear. Challenge in single tank array was done by anesthetizing the fish before adding 7–10 male and female pre-adult I lice directly to the skin of the fish. This procedure was conducted by placing the lice with their dorsal side on a small (around 5 × 5 cm) piece of wet paper. The paper was then turned around and attached to the skin on the dorsal part of the fish so that the ventral side of the louse was in direct contact with the fish. After careful removal of the paper, the correct number of lice was confirmed, and the fish were returned to the tank. Values in vaccinated and control groups were compared by Student’s *t*-test with Welch’s correction for unequal variances (*p* = 0.05, *n* = 10).

### 2.7. Salmon Lice Counts and Eggstring Sampling and Hatching

The number of lice (mainly chalimus II) was counted on each fish approximately 9 days after challenge with copepodids. For these counts, the fish were anesthetized with Tricain (PHARMAQ AS), and the whole surface of the fish was then inspected under a loupe. The fish was then returned to the tank and counted again approximately 3 weeks later when adult stages had developed. Effect sizes and *p*-values for the difference of means between vaccinated and control groups were calculated by Student’s *t*-test with Welch’s correction for unequal variances (*p* = 0.05, *n* = 35-40). For challenges in single tank arrays, the number of adults per fish was registered at the day of trial termination, and values in vaccinated and control groups were compared by Student’s *t*-test with Welch’s correction for unequal variances (*p* = 0.05, *n* = 10). Eggstrings were sampled from all adult females that had survived on the fish in single-tank arrays. First, the eggstrings were placed on a millimeter scale and photographed, so that the length could be measured. Then they were transferred to small plastic incubators with plankton netting and flow-through of water. Sets of eggstrings from individual lice were incubated in separate incubators. After approximately 2 weeks, the content of each incubator was fixated in 70% ethanol/30% seawater. The number of copepodids with normal morphology was then registered using a loupe for each incubator and compared to the length of the respective eggstrings. The success of hatching and molting to next generation copepodid was calculated as the number of normal copepodids per millimeter eggstring. For statistical analysis of eggstring and hatching results, the mean counts per fish in vaccinated and control groups were compared by the Mann–Whitney test (*p* = 0.05, *n* = 6–10).

### 2.8. ELISA for the Quantitation of Fish Antibody Titers

Antigen-specific antibody titers were determined in fish vaccinated in the single tank model. Serum was prepared from blood samples collected before salmon louse infestation, at approximately week 10 post-initial vaccination. For ELISA, high absorption capacity polystyrene microtiter plates were coated with 100 ng of the recombinant proteins (P21, P30, P33, and P37) per well in a carbonate–bicarbonate buffer (Sigma–Aldrich). After overnight incubation at 4 °C, coated plates were washed once with 100 µL/well PBS/0.05% Tween 20 (Sigma-Aldrich), then blocked with 100 µL /well of 5% bovine serum albumin (BSA) (Sigma–Aldrich) for 1 h at RT. Serum samples were diluted (1:100, *v*/*v*) in blocking solution, and 100 µl/well was added into the wells of the antigen-coated plates and incubated for 1.5 h at 37 °C. Plates were washed three times with PBS/0.05% Tween 20, and 100 µL/well of mouse anti-rainbow trout antibodies (Bio-Rad Laboratories, Inc.), diluted (1:1000, *v*/*v*) in blocking solution, was added and incubated for 1 h at RT. Plates were washed three times with 300 µl/well of PBS/0.05% Tween 20. A goat anti-mouse IgG-HPR conjugate (Sigma–Aldrich) was added (diluted 1:2000) in blocking solution and incubated for 1 h at RT. After four washes with 100 µL/well of PBS/0.05% Tween 20, 100 µL/well of 3,3′,5,5′-tetramethylbenzidine (TMB) one solution (Promega) was added and incubated for 15 min at RT. Finally, the reaction was stopped with 50 µL/well of 2 N H_2_SO_4_, and the optical density (O.D.) was measured in a spectrophotometer at 450 nm. Values in vaccinated and control groups were compared by Student’s *t*-test with Welch’s correction for unequal variances (*p* = 0.05, *n* = 10).

### 2.9. Analysis of Gene Expression by qRT-PCR

A real-time qRT-PCR was performed on RNA samples using gene-specific oligonucleotide forward (F) and reverse (R) 5′–3′ primers (P21F: CAACAGCATGCCTAGCAGAA and P21R: TCCATGCTTACAACCACCAA, P30F: ATTTCCGCATTTGACTACGC and P30R: GGCCAATTTCTTGGATCTGA, P33F: GTAACATTGCCTGGCCTCAT and P33R: TTCCAAGAGCTCGAACAGGT, P37F: TGCCGGTCAATTTCCATTAT and P37R: CATGTTTGCCTCGGAAGAAT, P14F: GCGAATCACGGAGTGGTACT and P14R: CCATGGAAACCCAATTTTTG), the Kapa SYBR Fast One-Step qRT-PCR Kit (Kapa Biosystems, Wilmington, MA, USA), and the Rotor-Gene Real-Time PCR Detection System (Qiagen Inc. Valencia, CA, USA). A dissociation curve was run at the end of the reaction to ensure that only one amplicon was formed and that the amplicons denatured consistently at the same temperature range for every sample. The mRNA levels were normalized against *elongation factor 1 α* (*EF1α*) as described previously [29] using the genNorm method (ddCT method as implemented by Bio-Rad iQ5 Standard Edition, Version 2.0) [30]. Normalized Ct values were compared between unfed and fed lice by Student’s *t*-test with unequal variance (*p* = 0.05; *n* = 3 biological replicates).

### 2.10. In Situ Hybridization

In situ hybridization (ISH) was used to demonstrate the localization of P33 in *L. salmonis*. The protocol applied is based on RNAscope technology using the RNAscope^®^ 2.0 HD Red Chromogenic Reagent Kit (Advanced Cell Diagnostics, Newark, CA, USA) and employing paired double-Z oligonucleotide probes targeting P33. The probes were designed using custom software as described by Wang et al. [31] to target nucleotides 3 to 337 of the P33 gene. Control probes (*dapB*, Advanced Cell Diagnostics Cat. No. 310043) targeting the bacterial *dapB* gene were used as negative control to assess background signals. The ISH was performed according to the manufacturer’s instructions. Briefly, formalin-fixed paraffin-embedded (FFPE) adult female *L. salmonis* sections were baked for 1 h at 60 °C prior to use. The baked sections were then deparaffinized in xylene and rehydrated through a standard series of alcohol washes. The rehydrated sections were incubated with hydrogen peroxide for 10 min at RT to block the endogenous peroxidases. The slides were then boiled in target retrieval buffer for 15 min before they were incubated with protease for 15 min at 40 °C. Then, a hybridization step with the probes specified above was performed at for 2 h 40 °C followed by a series of signal amplification and washing steps for 15 or 30 min at 40 °C. Finally, hybridization signals were developed by a chromogenic reaction using Fast Red substrate, and the slides were counterstained for 2 min using 1:1 (vol/vol) diluted Mayer’s hematoxylin 5B-535 (Chemi Teknik, Oslo, Norway).

### 2.11. Targeted Protein Identification

Precipitated plasma membranes were resuspended in 100 μL Laemmli sample buffer and applied onto 1.2-cm wide wells on a 12% SDS-PAGE gel. The electrophoretic run was stopped as soon as the front entered 3 mm into the resolving gel so that the whole proteome became concentrated in the stacking/resolving gel interface. The unseparated protein band was visualized by staining with GelCode Blue Stain Reagent (Thermo Scientific), excised, cut into 2 × 2 mm cubes, and digested overnight at 37 °C with 60 ng/μL sequencing grade trypsin (Promega, Madison, WI, USA) at 5:1 protein:trypsin (w/w) ratio in 50 mM ammonium bicarbonate, pH 8.8 containing 10% (*v*/*v*) acetonitrile [32]. The resulting tryptic peptides from the gel band were extracted by 30 min-incubation in 12 mM ammonium bicarbonate, pH 8.8. Trifluoroacetic acid was added to a final concentration of 1%, and the peptides were finally desalted onto OMIX Pipette tips C_18_ (Agilent Technologies, Santa Clara, CA, USA), dried-down and stored at −20 °C until mass spectrometry analysis.

The desalted protein digest was resuspended in 0.1% formic acid and analyzed by RP-LC-MS/MS using an Easy-nLC II system coupled to an ion trap LCQ Fleet mass spectrometer (Thermo Scientific). The peptides were concentrated (on-line) by reverse phase chromatography using a 0.1 mm × 20 mm C18 RP precolumn (Thermo Scientific) and then separated using a 0.075 mm × 100 mm C18 RP column (Thermo Scientific) operating at 0.3 μL/min. Peptides were eluted using a 180-min gradient from 5% to 35% solvent B (Solvent A: 0,1% formic acid in water, solvent B: 0.1% formic acid in acetonitrile). Electrospray ionization (ESI) was done using a Fused-silica PicoTip Emitter ID 10 μm (New Objective, Woburn, MA, USA) interface. Peptides were detected in survey scans from 400 to 1600 amu (1 μscan), followed by three data-dependent MS/MS scans (Top 3), using an isolation width of 2 mass-to-charge ratio units, normalized collision energy of 35%, and dynamic exclusion applied during 30 sec periods.

The MS/MS raw files were searched against a database containing the UniProt entries for the selected proteins (Table 1) (http://www.uniprot.org) using the SEQUEST algorithm (Proteome Discoverer 1.4, Thermo Scientific). The constraints imposed for the search were: tryptic cleavage after Arg and Lys, two maximum missed cleavages, tolerances of 20 ppm and 0.05 Da for precursor and MS/MS fragment ions, respectively, and Met oxidation and Cys carbamidomethylation as variable modifications. Searches were also performed against a decoy database in an integrated decoy approach. A false discovery rate (FDR) < 0.05 was considered as a condition for successful peptide assignments. The number of peptide-spectrum matches (PSMs) per protein was compared between fed and unfed louse membrane samples using a paired comparison Chi2-test (χ^2^) (*p* < 0.0001) in R software.

### 2.12. Immunofluorescence Assay

Rabbits were immunized subcutaneously with three 500 μL doses of recombinant P33 (50 μg/dose) in two-weeks intervals. Before immunization and two weeks after the third immunization, blood was collected, and IgGs were purified from the serum using a Montage Antibody Purification Kit and Spin Columns with PROSEP-A Media (Millipore, Billerica, MA, USA). The paraffin was removed from salmon louse sections with xylene and then hydrated by successive 2 min washes with a graded series of 100%, 95%, 80%, 75%, and 50% ethanol. The slides were treated with Proteinase K (Dako, Barcelona, Spain) for 7 min, washed with PBS, and incubated with 3% BSA (Sigma–Aldrich) in PBS for 1 h at RT. Then the slides were incubated with anti-P33 rabbit serum diluted 1:100 in 3% BSA/PBS for 14 h at 4 °C. After additional washes in PBS, the sections were incubated with FITC conjugated goat anti-rabbit IgG secondary antibodies (Sigma–Aldrich, St. Louis, MO, USA), diluted 1:80 in 3% BSA/PBS, for 1 h at RT. Finally, the slides were mounted using Prolong Gold antifade reagent with DAPI reagent (Molecular Probes, Eugene, OR, USA). The slides were examined using a Zeiss LSM 800 laser scanning confocal microscope (Carl Zeiss, Oberkochen, Germany). Sections incubated with pre-immune serum were used as controls.

## 3. Results

### 3.1. Salmon Louse Gut Membrane Proteins Overrepresented in Response to Feeding may Constitute Candidate Vaccine Protective Antigens

The main objective of this research was to identify candidate protective antigens for the control of salmon louse infestations in immunized salmon. Our hypothesis is that gut plasma membrane proteins overrepresented in fed lice when compared to unfed lice have potentially relevant biological functions for louse feeding and development and constitute good vaccine candidate protective antigens. The rationale behind this approach was to select antigens for which antibodies produced in immunized salmon will interact with the protein in feeding lice to affect its biological function and reduce ectoparasite feeding and development.

**Figure 1:**
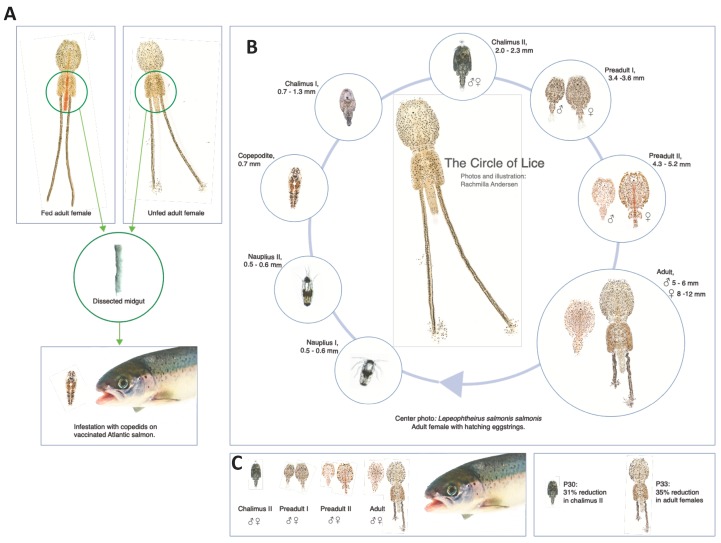
Experimental design. (**A**) Selected salmon louse membrane proteins (Table 1) were characterized in the midgut of fed and unfed adult female lice at both mRNA and protein levels by qRT-PCR, in situ hybridization, immunofluorescence assay (IFA) and targeted protein identification. Recombinant proteins were then evaluated in vaccine formulations for the immune-mediated reduction of salmon lice infestations in Atlantic salmon. Vaccinated Atlantic salmon were kept in the same tank as the control group and challenged with copepodids of *L. salmonis*. (**B**) Salmon lice lifecycle. (**C**) Numbers of lice per fish were counted when the majority of lice were at chalimus II stage and at the adult stage. The results showed a reduction in chalimus II and adult females in fish vaccinated with P30 and P33, respectively, when compared to controls. Photos and illustration: Rachmilla Andersen.

To test this hypothesis, an experimental approach was developed (Figure 1A–C) with selected salmon louse membrane proteins annotated at Uniprot (https://www.uniprot.org; updated May 2019) as involved in metabolism with various molecular functions (Table 1). The unsecreted cytoskeleton actin-binding protein moesin (P14; A0A0K2UFE6) was included as a negative control. These proteins were then characterized in the midgut of fed and unfed lice at both mRNA and protein levels by qRT-PCR, in situ hybridization, immunofluorescence assay (IFA), and targeted protein identification (Figure 1A). Then focusing on the salmon louse lifecycle (Figure 1B), recombinant proteins were evaluated in vaccine formulations for the immune-mediated reduction of salmon louse infestations (Figure 1A,C).

**Figure 2:**
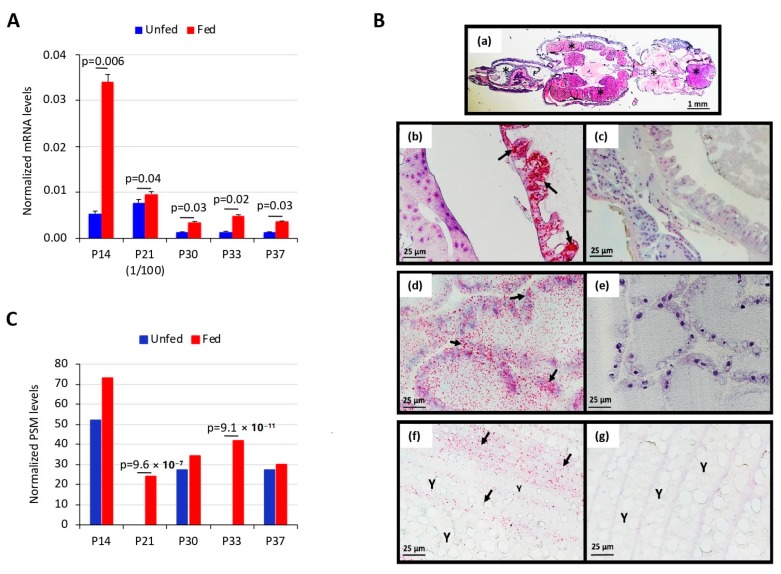
mRNA and protein levels of selected salmon louse membrane proteins. Selected salmon louse membrane proteins (Table 1) were characterized in the midgut of fed and unfed lice at mRNA and protein levels. (**A**) The qRT-PCR was performed on RNA samples using gene-specific oligonucleotide primers. The mRNA levels were normalized against *eEF1α*. Normalized Ct values were compared between unfed and fed lice by Student’s *t*-test with unequal variance (*p* = 0.05; *n* = 3 biological replicates). (**B**) Demonstration of *L. salmonis* P33 transcripts in sections of adult female salmon louse using in situ hybridization. (a) Light microscopy (1.25X/0.04 lens) of hematoxylin stained whole mature female *L. salmonis* where asterisks indicate the tissue compartments, which generally were strongly stained for P33 transcripts by in situ hybridization (ISH). (b–g) RNAscope ISH of adult female *L. salmonis* FFPE sections using the following probes: P33-specific probes (b,d,f) and *dapB* negative control probes (c,e,g). Specific staining for P33 is seen as red-color dots. (b) Staining for P33 is observed in the alimentary tract, predominantly confined to intestinal epithelium (arrows). (d) Staining for P33 is widespread in the ovarian tubules but particularly present in cells associated with the ovarian tubule membrane (arrows). (f) In the vitellogenic oocytes, staining for P33 is predominantly confined to the cellular rim of the individual oocytes (arrows), but not in egg yolk (Y). Photos in panels (b–g) were taken with a 40X objective lens. (**C**) Plasma membrane protein levels were analyzed by RP-LC-MS/MS. The MS/MS raw files were searched against a database containing the UniProt entries for the selected proteins (Table 1). A false discovery rate (FDR) < 0.05 was considered as a condition for successful peptide assignments. The number of peptide-spectrum matches (PSMs) per protein was compared between fed and unfed louse membrane samples using a paired comparison Chi2-test (χ^2^) (*p* < 0.0001) in R software.

The expression of selected candidate protective antigens (Table 1) was first characterized by qRT-PCR, and the results showed significant upregulation (*p* < 0.05) in response to salmon louse feeding with the highest mRNA levels for P21 (Figure 2A). RNAscope in situ hybridization was then used to demonstrate P33 transcripts in FFPE sections of *L. salmonis* (Figure 2B). Positive staining related to P33 gene expression was identified as red punctate dots that were present in the sections hybridized with the P33-specific probe but not in those hybridized with the negative control probe (Figure 2B). In adult female salmon lice, expression of P33 transcripts was clearly shown in the alimentary tract, the ovaries in the cephalothorax, and the vitellogenic oocytes in the genital segment. In the alimentary tract, the staining was diffusely distributed, likely in the cytoplasm of intestinal epithelial cells (Figure 2(Bb)), and the staining intensity tended to vary along the length of the tract. In the ovaries, staining was seen widespread in the convoluted ovarian tubules, but with a certain focus in cells associated with the ovarian tubule membrane (Figure 2(Bd)). In the vitellogenic oocytes, staining was predominantly confined to the cellular rim of the eggs, while the yolk in the center was negative (Figure 2(Bf)).

**Figure 3:**
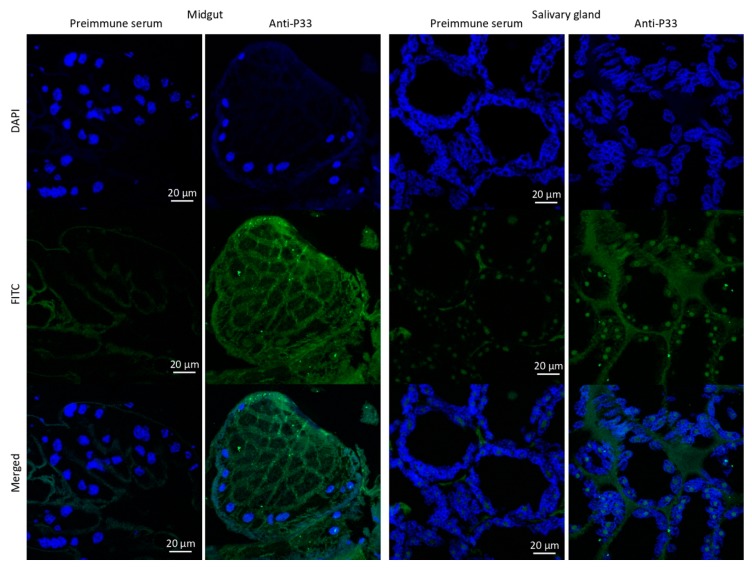
Localization of P33 in salmon louse midgut and salivary glands. The green channel is P33 stained with goat anti-rabbit IgG-FITC antibodies, and the blue channel is DAPI-stained DNA (nucleus). Merged combine images of green and blue channels. There was no specific labeling of P33 in negative control sections treated with preimmune serum. Scale bar: 20 µm.

At the protein level, targeted protein identification demonstrated significant overrepresentation (*p* < 0.0001) of P21 and P33 in response to louse feeding and in agreement with results at the mRNA level (Figure 2C). Finally, the IFA of salmon louse tissues showed the presence of protein P33 in both midgut and salivary glands (Figure 3).

### 3.2. Vaccination of Atlantic Salmon with Selected Salmon Louse Midgut Proteins Affects Ectoparasite Infestations

**Figure 4:**
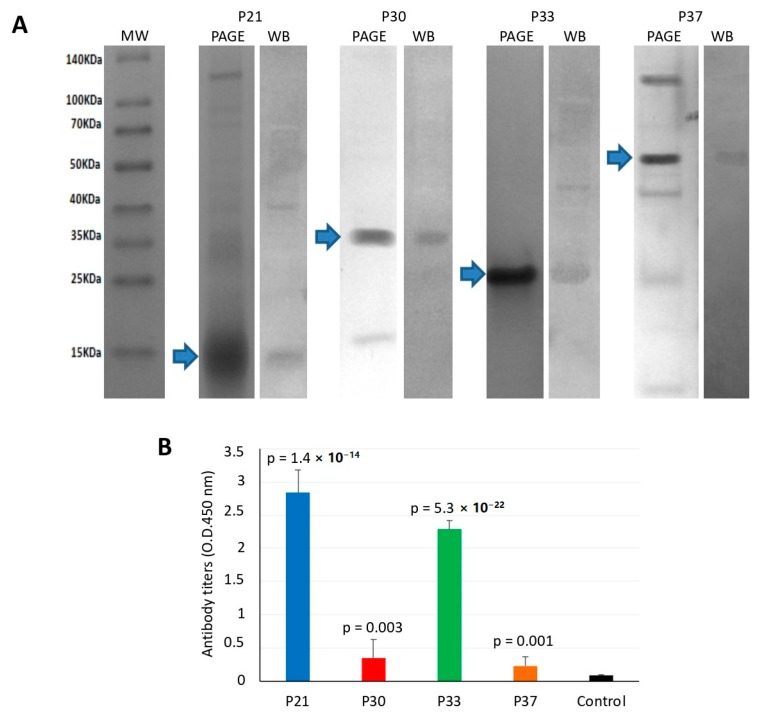
Antibody response in vaccinated fish. (**A**) The recombinant proteins were produced in *E. coli* and analyzed by polyacrylamide gel electrophoresis (PAGE) and Western blot (WB). Ten micrograms of recombinant proteins was loaded onto a 12% SDS-polyacrylamide gel and stained with Coomassie Brilliant Blue or transferred to a nitrocellulose membrane. For Western blot analysis, pooled sera collected from vaccinated salmons before salmon louse infestation at approximately week 10 post-initial vaccination were used as primary antibodies. The membrane was then incubated with mouse anti-rainbow trout antibodies and revealed with a goat anti-mouse IgG-HPR conjugate. (**B**) Antibody titers were determined in vaccinated and control fish by ELISA. Values of D.D. at 450 nm in vaccinated and control groups were compared by Student’s *t*-test with Welch’s correction for unequal variances (*p* = 0.05).

Recombinant proteins were produced in *E. coli*, purified, and used for vaccine formulation (Figure 4A). Vaccination trials with sea lice midgut proteins were conducted in common and single tanks. Antibodies produced in response to vaccination were antigen-specific, as shown by Western blot analysis of fish sera (Figure 4A). Antibody titers to vaccination antigens were determined in fish from the single tank model, which were higher in vaccinated than control fish (Figure 4B). The highest antibody titers were obtained in fish vaccinated with P21 and P33 (Figure 4B).

Challenges done with infectious salmon lice in a common tank model demonstrated differences in lice levels for fish vaccinated with P33 and P30, but not for the other antigens (Table 2). The best protection was observed for P33, with a 35% reduction of adult female lice when compared to the control group (*p* = 0.004; Table 2). For P30, a reduction of 31% was observed at the chalimus stage when compared to controls (*p* = 0.015; Table 2).

Eggstrings produced by lice that fed on vaccinated fish in single tank arrays were, on average, shorter than eggstrings originating from lice that fed on control fish for all groups except P37 (Table 3). However, these differences were significant only for P30 (16% reduction, *p* = 0.042) but not for the other antigens. Eggstrings from all groups hatched normally with no significant differences between groups (Table 3). The number of adult females that developed in single tank arrays did not differ significantly between vaccinated and control groups (Table 4). However, the statistical power for this analysis was low in this model and only suitable to detect rather large effects. Nevertheless, a tendency was observed towards a reduction in both adult females and males in fish vaccinated with P33 and P30 (Table 4).

## 4. Discussion

The rational approach for the identification of candidate protective antigens was proposed and successfully applied in ticks [26,37]. Focusing on functionally relevant biological processes and proteins, such as tick attachment and feeding, proteases and protease inhibitors at the tick-host interface, water balance, blood digestion, heme and iron metabolism, detoxification, mating, vitellogenesis, and fertility, allowed the identification of candidate vaccine tick protective antigens [26,37]. Some of these antigens, such as Aquaporin [37,38,39,40], Ferritin [41,42,43,44], and 64TRP cement protein [45,46], have shown efficacy in vaccine formulations for the control of tick infestations, thus providing support for the potential of this predictive model for the identification of candidate protective antigens. Nevertheless, these experiments were conducted in mammalian animal models, which show differences in the immune response when compared to fish [47].

In this study, we identified four candidate salmon louse protective antigens by using the rational approach focused on midgut function and blood digestion (Table 1). Two of the candidate protective antigens, P33 and P30, resulted in a significant reduction of sea lice infestations in vaccinated fish by reducing adult female (P33) or chalimus II (P30) infestations (Figure 1C). Toll-like receptors (TLRs), such as TLR6 (P30), play an important role in the innate and adaptive immune responses to pathogens and are the target of new vaccine adjuvants by using lipopeptides that activate TLR2/6 heterodimers for use in *Leishmania* vaccines [48]. Potassium chloride and amino acid transporters and other solute carriers involved in cellular hypotonic salinity response and transmembrane transport, such as P33, have been used as candidate protective antigens in vaccine formulations against hard ticks (e.g., Aquaporin; [37,38,39,40]), soft ticks (e.g., ABC transporter; [37] and sulfate/anion exchanger; [49]) ticks and cat flea (e.g., zinc transporter ZIP13 homolog; [25]). Solute carriers, such as human solute carrier family 11 member 1 protein (SLC11A1), have been implicated in the control of bacterial replication and might influence the responses to vaccines with recombinant bacteria in different ways such as regulation of bacterial load or recombinant antigen dose, class II molecule expression, costimulatory or adjuvant activity, or antigen processing [50].

Our results supported the selection of salmon louse P30 and P33 as candidate vaccine protective antigens for the control of ectoparasite infestations. The model used in this study is valid, but the observed vaccine efficacy was moderate for these antigens, and probably on the lower end of what would be acceptable for commercial use. Nevertheless, the true vaccine efficacy is difficult to ascertain for sea lice vaccines in laboratory trials due to challenges with reproducing a coherent life cycle of the parasite on one host. First, the lice tend to jump between hosts kept in the same tank when they reach the preadult I stage, thus creating noise when studying motile life stages in common tank setups and may disguise effects of vaccination [51]. Second, the development of next generation copepodids from nauplia happens in the pelagic phase and must, therefore, be done in vitro.

Based on studies conducted with other ectoparasites (i.e., [25,52,53]), the hypothesis is that the observed effects of vaccination with P33 and P30 antigens is caused by binding of specific IgM antibodies to the target protein present on intestinal cell membranes and thus affecting protein function resulting in the reduction of sea louse viability.

## 5. Conclusions

In summary, the results of this study showed that the translational approach allowed the identification of candidate protective antigens in salmon louse. Two of these antigens, P30 and P33, provided protection against salmon lice infestation in Atlantic salmon by reducing adult female (P33) or chalimus II (P30) infestations.

## 6. Patents

The results of this study were part of the patent application by de la Fuente Garcia, J., Contreras Rojo, M., Villar Rayo, M.M, De Feijter Karlsen, M.A., Brudeseth, B.E., Lindmo Yttredal, K., Wiik-Nielsen, C.R., Olsen, R.H., Hungerholdt, L.B. Sea Lice Vaccine. Filing date: August 16, 2017. PCT/US2017/047095. Publication number: WO/2018/035199 (22.02.2018). https://patentscope2.wipo.int/search/en/detail.jsf?docId=WO2018035199.

## Figures and Tables

**Table 1:** Proteins selected as candidate vaccine targets.

Protein: Accession No.	Description	GO	References
P21: A0A0K2VDM5	Delta-like protein	BP: Metabolism, multicellular organism development, Notch signaling pathway MF: Calcium ion binding CC: Membrane	[33,34]
P30: A0A0K2T2M9	Putative Toll-like receptor 6	BP: Metabolism, signal transduction, innate immune response MF: Receptor CC: Membrane	[35]
P33: A0A0K2TQ92	Potassium chloride and amino acid transporter	BP: Metabolism, amino acid transmembrane transport, Cellular hypotonic salinity response MF: Transmembrane transporter activity, Potassium:chloride symporter activity CC: Membrane	[36]
P37: A0A0K2UYH4	Bifunctional heparan sulfate *N*-deacetylase/*N*-sulfotransferase	BP: Metabolism, cell surface receptor signaling pathway, Synaptic vesicle endocytosis, Wnt signaling pathway MF: [heparan sulfate]-glucosamine *N*-sulfotransferase activity CC: Membrane	[34]

Proteins were annotated at Uniprot (https://www.uniprot.org; updated May 2019) and considering amino acid sequence homology in other organisms. Abbreviations: BP, biological process; MF, molecular functions; CC, cell compartment.

**Table 2:** Effect of vaccination on sea lice infestations in the common tank model.

Antigen	Fish (*n*)	Chalimus II	Adult Male	Adult Female	Reduction of Chalimus II	Reduction of Adult Females
P33	36	13.64 (12.05–15.32)	4.17 (3.36–4.98)	3.81 (3.12–4.49)	14% *p* = 0.12	35% *p* = 0.004
Control P33	40	15.85 (13.58–18.12)	4.95 (4.14–5.76)	5.83 (4.66–6.99)		
P30	35	12.14 (9.85–14.44)	2.66 (1.80–3.52)	1.17 (0.58–1.76)	31% *p* = 0.02	25% *p* = 0.30
Control P30	40	17.56 (13.94–21.21)	2.67 (2.05–3.28)	1.56 (1.07–2.06)		
P37	40	27.98 (24.78–31.17)	8.46 (7.10–9.56)	8.00 (6.53–9.47)	7% *p* = 0.28	4% *p* = 0.69
Control P37	40	30.20 (27.51-32.88)	8.02 (6.87–9.17)	8.37 (7.17–9.56)		
P21	39	19.72 (16.48-22.96)	1.31 (0.96–1.66)	0.13 (0.04–0.30)	20% *p* = 0.09	0%
Control P21	40	24.50 (19.81–29.19)	1.18 (0.81–1.54)	0.13 (0.02–0.23)		
P14	40	5.05 (3.99–6.11)	2.42 (1.73–3.22)	2.39 (1.79–2.99)	12% *p* = 0.40	0%
Control P14	40	5.71 (4.55–6.87)	2.47 (1.85–2.99)	2.39 (1.71–3.07)		

Vaccinated Atlantic salmon were kept in the same tank as the control group and challenged with copepodids of *L. salmonis*. Numbers of lice per fish were counted when the majority of lice were at chalimus II stage and at adult stage. Means for each group with 95% confidence intervals are shown. Values in vaccinated and control groups were compared by Student’s *t*-test with Welch’s correction for unequal variances (*p* = 0.05). The effect on males was not calculated because their contribution to fecundity is unclear.

**Table 3:** Effect of vaccination on sea lice eggstring length and hatching in the single tank model.

Antigen	Fish (*n*)	Eggstring Length (mm)	Copepodids/mm Eggstring	Reduction of Eggstring Length	Reduction of Hatching Success
P33	10	9.23 (7.57–10.89)	10.59 (7.66–13.52)	15% *p* = 0.09	1% *p* = 0.74
Control P33	10	10.82 (9.63–12.00)	10.74 (8.09–13.38)		
P30	6	12.63 (10.48–14.78)	8.28 (5.47–11.08)	16% *p* = 0.04	−13% *p* = 0.49
Control P30	8	15.08 (14.25–15.91)	7.36 (4.50–10.21)		
P37	10	17.15 (16.24–18.05)	10.10 (8.38–11.83)	-5% *p* = 0.40	−44% *p* = 0.17
Control P37	10	16.40 (15.00–17.80)	7.01 (3.81–10.21)		
P21	8	6.69 (2.69–10.69)	5.05 (-0.87–10.97)	24% *p* = 0.42	−44% *p* = 0.70
Control P21	7	8.79 (4.58–12.99)	3.50 (-2.20–9.19)		
P14	9	13.94 (9.86–18.03)	7.99 (4.60–11.39)	7% *p* = 0.54	−9% *p* = 0.52
Control P14	8	15.00 (11.32–18.67)	7.35 (4.17–10.54)		

Eggstrings were collected from adult females that originated from vaccinated and control fish-bearing gravid female lice in single tanks. Successful hatching was measured by counting the number of copepodids that developed from each set of eggstrings. Means for each group with 95% confidence intervals are given. Values in vaccinated and control groups were compared by the Mann–Whitney test (*p* = 0.05).

**Table 4:** Effect of vaccination on sea lice infestations in the single tank model.

Antigen	Number of Pre-Adults at Challenge		Number of Adults at Termination		Reduction	
	Males	Females	Males	Females	Adult Males	Adult Females
P33	7.30 (±0.62)	6.90 (±0.19)	0.90 (±0.43)	2.80 (±0.61)	50% *p* = 0.07	13% *p* = 0.45
Control P33	7.40 (±0.50)	7.00 (±0.00)	1.80 (±0.82)	3.30 (±0.88)		
P30	6.70 (±0.30)	8.00 (±0.00)	0.80 (±0.46)	1.20 (±0.67)	27 % *p* = 0.43	29% *p* = 0.38
Control P30	6.80 (±0.25)	8.00 (±0.00)	1.10 (±0.51)	1.70 (±0.80)		
P37	10.00 (±0.00)	10.00 (±0.00)	1.70 (±0.68)	4.30 (±1.08)	32% *p* = 0.16	-10% *p* = 0.60
Control P37	10.00 (±0.00)	10.00 (±0.00)	2.50 (±0.75)	3.90 (±0.90)		
P21	7.00 (±0.00)	8.00 (±0.00)	0.20 (±0.25)	1.30 (±0.56)	−100% *p* = 0.56	−44% *p* = 0.31
Control P21	7.00 (±0.00)	8.00 (±0.00)	0.10 (±0.19)	0.90 (±0.43)		
P14	10.00 (±0.00)	10.00 (±0.00)	1.20 (±0.67)	2.80 (±1.03)	−33% *p* = 0.52	−87% *p* = 0.07
Control P14	10.00 (±0.00)	10.00 (±0.00)	0.90 (±0.51)	1.50 (±0.69)		

Vaccinated Atlantic salmon (*n* = 10 per group) were kept in single tanks (one individual per tank) and challenged with pre-adults of *L. salmonis*. Numbers of lice per fish were counted when lice reached adult stages. Means for each group with 95% confidence intervals are shown. Values in vaccinated and control groups were compared by Student’s *t*-test with Welch’s correction for unequal variances (*p* = 0.05).
